# Private and public health care in rural areas of Uganda

**DOI:** 10.1186/1472-698X-10-29

**Published:** 2010-11-24

**Authors:** Joseph Konde-Lule, Sheba N Gitta, Anne Lindfors, Sam Okuonzi, Virgil ON Onama, Birger C Forsberg

**Affiliations:** 1 Department of Epidemiology and Biostatistics, School of Public Health, Makerere University, P.O. Box 7072, Kampala, Uganda; 2 Division of Global Health (Ihcar), Dept of Public Health Sciences, Karolinska Institutet, S-171 77 Stockholm, Sweden; 3 Regional Center for Quality of Health Care, School of Public Health, Makerere University, P.O. Box 7072, Kampala, Uganda; 4 Health Policy Planning and Management, School of Public Health, Makerere University, P.O. Box 7072, Kampala, Uganda

## Abstract

**Background:**

In many low and middle income countries, the private sector is increasingly becoming an important source of health care, filling gaps where no or little public health care is available. However, knowledge on the private sector providers is limited The objective of this study was to determine the type and number of different types of health care providers, and the quality, cost and utilization of care delivered by those providers in rural Uganda.

**Methods:**

The study was carried out in three rural districts. Methods included (1) mapping of health care providers; (2) a household survey to determine morbidity and health care utilization; (3) a health facility survey to assess quality of care; (4) focus group discussions to get qualitative information on providers and provider choice; and (5) key informant interviews to further explore service characteristics.

**Results:**

95.7% of all 445 facilities surveyed were private while 4.3% were public. Traditional practitioners and general merchandise shops that sold medicines comprised 77.1% of all providers. They had limited infrastructure and skills but were often located in the villages and therefore easily accessible. Among the formal providers there were 4 times as many private for profit providers than public, 76 versus 18. However, most of the private units were one-person drug shops.

In the household survey, 2580 persons were interviewed. 1097 (42%) had experienced illness during the preceding month. Care was sought in 54.1% of the cases. 35.6% were given self-treatment and in 10.3% no action was taken. Of the episodes for which people sought care at a health care facility, 37.0% visited a public health care provider, 39.7% a for profit provider, 11.8% a private not for profit provider, and 10.6% a traditional practitioner. Private for profit facilities were the most popular for ambulatory health care, while public facilities were preferred for more serious conditions and for hospitalization. Traditional practitioners were many but saw relatively few patients. They were mostly used for social problems and limited medical specific conditions.

**Conclusions:**

Private providers play a major role in health care delivery in rural Uganda; reaching a wide client base. Traditional practitioners are many but have as much a social as a medical function in the community. The significance of the private health care sector points to the need to establish a policy that addresses quality and affordability issues and creates a strong regulatory environment for private practice in sub-Saharan Africa.

## Background

In many low and middle income countries, the private sector is increasingly becoming an important source of health care, filling gaps where no or little public health care is available [1]. Private providers are also often the main source of health care even in places where free or low cost public services are at hand [2-5]. In Nigeria, private maternity centers are the most preferred health care facilities for child birth, followed by traditional birth attendants [6]. In India, 60-80% of all TB cases initially seek care from private providers, and two thirds of those continue with private providers rather than seek care at a public provider after getting diagnosed [7]. In Bangladesh, 80% of all consultations for childhood diarrhoea take place with private providers [8].

Like many other countries [9-12], Uganda is at the stage of promoting and even formalizing linkages between public and private health care systems with the aim of improving access to health care [13,14]. However, knowledge on the private sector providers is limited and this makes it difficult to include them in health care planning. Access to health care is an important area of study in most developing countries, including Uganda. Reaching the hitherto un-reached has gained increasing priority, in part due to the global movement for attaining the Millennium Development Goals (MDG). The extreme range of health care providers in the region, all with different levels of training, skills and capabilities, offering services of diverse quality makes the task of studying them all difficult. Most studies have therefore commonly tended to target specific categories of health providers, mostly public, rather than looking at the full spectrum of providers. Hence, in order to expand the knowledge base for planning and policy making there is a need to identify all types of health care providers in the community and to quantify the fraction of clients that each type attends to.

The overall objective of this study was therefore to determine the scope and character of health care services in rural areas in Uganda with a particular focus on private services. More specifically, the study looked at the frequency of different types of health care providers in rural Uganda, the quality of care they offer and their utilization.

## Methods

This study was conducted in three rural Ugandan districts, Iganga, Mpigi and Masaka, as part of a larger study to assess the potential of the private sector to improve health outcomes in Uganda. The three districts were purposefully selected as typical districts to represent eastern, southwest and central Uganda where the large majority of the Ugandan population lives. The research strategies included mapping of all health care providers in defined areas, a household survey, a health facility survey, focus group discussions in the community and key informant interviews with selected stakeholders in the health sector.

### Household survey and mapping of health care providers

The household survey and the mapping of health care providers were done concurrently in the same communities. In each selected district, three sub-counties were purposively selected to be representative of the rural-urban divide of the district: One sub-county had to be adjacent to the Town Council (this was considered to be urban), one remote sub-county and finally the third one was located midway between the remote and the urban sub-counties. In each sub-county two parishes and in each parish two villages were randomly selected.

A mapping was conducted to list all available health units from the 18 parishes, including public and private providers. The latter included for profit, not for profit, formal, informal, allopathic and traditional health care providers. All health care providers acknowledged by local community representatives as a regular provider were mapped. The mapping included recording of GPS coordinates. The GPS data enabled generation of maps showing the distribution of different providers in the mapped areas. General merchandise shops that sold medicines to the public were also included in the mapping. "Mobile providers", who travel through villages and market places with treatment kits, were however excluded.

For the household survey, the study team identified a starting point in each village from a list of households obtained from the village chairman. After identifying the first household, every fifth household was enrolled into the study until 12 households were enrolled. All persons 15 years and older were interviewed. For children below 15 years, responses were sought from an adult familiar with the child's health. Face to face interviews were conducted by research assistants to elicit health seeking behaviors for any illness in the past 30 days and then for serious conditions over the previous 12 months. Households that could not be included due to absence or other reasons were replaced by the household with the threshold nearest to the skipped household.

The study team recruited research assistants with experience in conducting surveys. They were formed into three interviewer teams, each comprising 4 interviewers and 1 supervisor. The investigators conducted training of the research teams for a period of 1 month. The interviewer teams were walked through the objectives, methods and tools of the study to enable them to grasp the details of the study requirements. Subsequently, explanations, question and answer sessions and mock interviews were held. After completion of the training of the research teams, the instruments of the household survey were pre-tested by the study and research teams in Nangabo sub-county, a rural area in Kampala district.

Once in the field, the supervisors edited completed questionnaires before the interviewers left the field. At the end of each day, the supervisors together with their interviewers reviewed the data collected to correct mistakes and look out for missing data. The supervisors ensured that when data were missing the interviewer returned to the households with the supervisor to collect the missing information.

Quantitative data was entered and analyzed in Epi-info 2002. The data was tested for normality and transformation was done where necessary. Odds ratios, chi-square and t-tests were used to test for significant associations. Qualitative data was analyzed manually using the master sheet technique - individual responses were coded with respect to respondents. Ethical approval was received from Makerere University Institute of Public Health Higher Degrees Research and Ethics Committee and informed written or verbal consent was obtained from each respondent.

### Focus group discussions

Focus group discussions were conducted after completion of the household survey to obtain explanations to unexpected responses and to enrich the data. In each sub-county 4 focus groups were held, two with community leaders and two with purposefully selected traditional practitioners. In total 36 focus groups discussions were held, each one with on average 8 participants. Most results from the focus groups are reported elsewhere.

### Key Informant Interviews

Key informant interviews were held with community leaders, like council members in the local community, district health administrators in the districts and with policy makers at the national Ministry of Health headquarters.

### Health facility survey

A health facility survey of formal facilities was conducted of 22 public and 60 private facilities. The facilities were sampled from the whole study area. The 22 public facilities included all public facilities in the study area. The 60 private providers included all 13 Private Not for Profit facilities (PFNP) and a one in three sample of the 76 Private for Profit (PFP) and 43 general merchandise shops that sold drugs in the study area. Quality of care was assessed by recording the qualifications of the providers, infrastructure and diagnostic equipments available, range of services offered and types of diseases handled. Infrastructure was assessed for presence of sterilizer, examination bed and treatment spaces. The diagnostic equipments for which availability were checked for were thermometer, stethoscope, blood pressure machine and weighing scale. Number and qualification of medically trained personnel was also recorded. Clinical competence was assessed using case scenarios, where a provider was presented with a specific case scenario and his/her approach to diagnosis, investigations, treatment and advice including referral was evaluated against the national treatment guidelines also known as Uganda Clinical Guidelines (UCG) from the Ministry of Health. Common disease presentations were used including fever, acute respiratory illnesses and diarrhoeal disease among children and diabetes mellitus among adults as case scenarios. The required answer included a diagnosis and appropriate treatment.

## Results

### Availability

The facilities (total n = 445) mapped by category were:

• 19 public facilities (1 hospital and 18 health care centers),

• 7 Private Not For Profit (PNFP) facilities *(*all health care centers);

• 76 Private For Profit (PFP) facilities,

• 300 traditional practitioners comprising herbalists, bone setters and spiritualists and

• 43 general merchandise shops, selling medicines or condoms in the mapped area.

**Figure 1:**
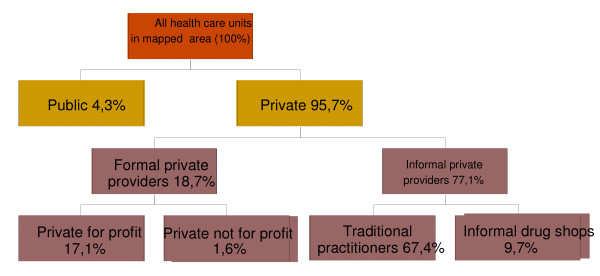
Relative frequency of health care facilities by type of provider

Figure 1 shows the proportion of the different health care units that were mapped.

The public facilities made up only 4.3% of all the listed facilities while 95.7% were private. Of the 95.7% private facilities only 18.7% were formal facilities while 77.1% were informal. Hence, informal providers, defined as unqualified providers without formal training, were the most numerous providers in the mapped areas. Private for profit clinics and drug shops made up 17.1% of all mapped facilities while the PNFP sector contributed 1.6% of all mapped facilities.

We found that most of the informal providers were one-person ventures, offering a narrow range of services on an irregular basis. During the household survey and focus groups, it was later found that the role of informal providers was relatively limited. When informal units are excluded the distribution of facilities is summarized in table 1.

**Table 1:** Formal health facilities in mapped areas in three districts in Uganda

*District*	*Public*	*PNFP*	*PFP*	*Total*
Masaka	5	3	44	52
Mpigi	9	2	18	29
Iganga	5	2	14	21
*Total*	*19*	*7*	*76*	*102*
**Percentage**	**18.6**	**6.9**	**74.5**	**100**

The government aims to have at least one public or PNFP facility in every parish. This target was realized in the mapped areas. These two categories of health facilities combined, however, made up only 25% of all the mapped formal facilities while private for profit providers stood for 75% of all available formal health care facilities. Among the formal providers, the private for profit category is therefore the most common type of provider available to rural communities.

### Utilization

In the household survey, 2580 persons were interviewed and 42% (1097) reported that they experienced illness once or more during the previous 30 days, giving a total of 1269 disease episodes. Of the 1269 disease episodes, the ill person sought care from a health care facility in 54.1% of the cases (687/1269), while in 35.6% (452/1269) of the episodes, the illness was treated by themselves and in 10.3% (130/1269) of the cases nothing was done about the illness (table 2).

**Table 2:** Actions taken when falling ill during 1269 illness episodes in 1097 patients interviewed in the household survey

*Action taken/where care was sought from*	*Number (N = 1269)*	*Percentage*
Did nothing	130	10.3
Self treated	452	35.6
Public health units	254	20.0
Private not for profit (PNFP)	81	6.4
Private for profit (PFP)	273	21.5
Traditional practitioners	73	5.8
Other	6	0.5
Total treatment episodes	1269	100

Of the disease episodes for which people sought care at a health care facility (n = 687), 37% visited a public health care provider, 11.8% a PNFP, 40% a PFP and 10.6% a traditional practitioner. Hence, 63% of the patients visited private providers and 37% public. Overall, the private for profit facilities were the most popular for ambulatory health care, while public facilities were preferred for more serious conditions and for hospitalization.

Data on socio-economic status showed that out of the 193 sick peasants that sought care, 100 (52%) sought care from a public provider while among traders only 38% went to public facilities. This difference is statistically significant (odds ratio [OR] = 1.88, 95% confidence interval [CI] = 1.02 - 3.49, and p value, p = 0.03). Adults (18+ years) who had primary education as highest level of education were less likely to seek care from a health care provider than those with secondary and tertiary education (52% versus 64%). This difference is statistically significant ([OR] = 0.602, 95% [CI] = 0.38-0.954, p = 0.03).

### Taking no action to treat the illness

Approximately 10.3% (130/1269) of respondents said that they did nothing about their illness. The main reason given for this was the perception that the problem was minor (58.5%; 76/130). Other reasons were "no money" (37.7%; 49/130), "no good care available" (36.2%; 47/130), "long distance" (10%; 13/130) and "no transport" (3.1%; 4/130).

### Self-treatment

Self treatment is when a person decides what medicine to take without advice from a health worker. Even when medicine is purchased from a drug shop or pharmacy it remains self treatment if he or she decides what to buy and how to use it. The fraction of persons that tried self-medication was 35.6% (452/1269). Of these, 60.4% (273/452) used medicine that was available at home and 50% (226/452) used home-made medicines including roots, oils and herbs (several actions allowed). Among those who self-treated and were 10 years or older at the time of the survey, 59.7% (267/447) reported to have missed work or school due to the illness.

### The role of traditional practitioners

When all the respondents were asked whether they had at any time visited a traditional practitioner for the health problem under investigation, only 5.8% (73/1269) reported having done so. This proportion is relatively small in comparison with the mapping, which showed that 67.4% of all available health care units belonged to traditional practitioners.

From the focus groups and site visits it was found that traditional practitioners are not consulted for the commonest illnesses such as fever or cough. They tend to be approached for specific conditions at which their competence is recognized, mainly social problems including family relations and wealth seeking. Also among those who provide treatment, many can only treat one type of medical condition such as fractures, child birth, impotence or snake bites. Such disease-specific practitioners may spend long periods of time, commonly many weeks or months, without treating even one patient. While a few of them also treat some of the common illnesses, there was a general view from the focus group discussions that most traditional practitioners are more of social workers than medical workers.

The traditional practitioners that participated in the focus group discussions said that a large proportion of their clients consult at night, under the cover of darkness to avoid being seen. They also reported that many of their clients come from distant places because a majority of patients prefer to visit healers outside their areas of residence to further conceal their identity. This view was also echoed by community leaders that participated in the focus group discussions. Those findings indicate that stigma at times is linked with traditional practitioner consultations. Hence, there may be some under-reporting in the household survey on visits to this provider group.

### Choice of provider

When asking the participants in the household survey why they choose to go to the particular provider, proximity, skills of the provider and low cost were the most common reasons given. A comparison of reasons among persons that visited different providers is summarized in table 3.

**Table 3:** Reasons for choosing a health care provider among households surveyed in three districts in Uganda sorted by type of provider

	Reason for choice of provider			
	
*Type of provider visited*	*Number *	*Proximity No. (%)*	*Skills No. (%)*	*Low cost No. (%)*
Public	251	103 (41.0)	113 (45.0)	29 (11.6)
PNFP	80	45 (56.3)	33 (41.3)	8 (10.0)
PFP	269	159 (59.1)	71 (26.4)	30 (11.2)
Traditional Practitioner	73	18 (22.8)	27 (34.2)	15 (19.0)
General merchandise shop	6	5 (83.0)	0 (0%)	1 (17.0)

In public units the most common reason for choice was technical skills (45%), closely followed by proximity at 41%. The leading reason for visiting the PNFP was proximity (56.3%), followed by skills (41.3%). In the PFP category the leading reasons for choice was proximity (59.1%) and skills (26.4%).

### Quality of Care

The quality of care was assessed on the basis of existing infrastructures, trained staff, equipment and good clinical skills of provider. The quality of care for all public and PNFP facilities was found to be good or satisfactory. Most PFP units were also assessed to have satisfactory quality of care but some few, especially clinics or drug shops that were manned by persons that were not fully trained, were assessed to have unsatisfactory quality of care. This is because many of them were short on space, lacked the basic diagnostic equipments like thermometer or blood pressure machine and failed the clinical competence tests. The assessment tool scored the informal units very low because none of them had basic equipment.

## Discussion

In this study we have described the scope and practices of private providers in three districts in Uganda. Though health service utilization in Uganda has been studied and discussed frequently earlier [15], this study is the first comprehensive description of the total private sector in rural districts in Uganda. The significant role of the private sector in health care delivery in Uganda has been documented by the study through a thorough mapping and a survey of providers.

Private providers outnumbered by far the public providers and a majority of the persons interviewed in the community chose to turn to private providers when seeking care. Though the number and distribution of public providers in the mapped areas fulfilled the government criteria for access to service supply, use of private providers was still considerably higher than use of public facilities. This may seem surprising as public services officially are free in Uganda. However, as documented by McPake et al. informal fees are levied by many health workers in the public facilities [16]. Other studies have documented poor quality of public services [17]. These factors may in combination contribute to many people's preferences for private care.

The findings of the study are of immediate relevance to public health advancement. The potential of the private sector to contribute to public health services and health improvement has been documented for HIV/AIDS and malaria control in Uganda [18,19] and the scope of the private sector as indicated by this study should result in more active engagement of the private sector in public health promotion.

In this study it was found that proximity was the most common reason for choosing a private provider when falling ill. Since private providers are more frequent in the community they have a higher probability of being the closest care provider, a fact that in itself can explain some of the higher use of private services. When private providers are the closest, transportation and other non-medical expenses appear to raise barriers to seeking publicly provided health care [20].

Except from a convenient location, perceived high technical skills of the personnel has also been shown to be an important reason for patients' preferences for private practitioners [15]. In this study, skills were the most common reason for choosing PNFP provider or a public provider. However, only 26% of the clients of PFP stated that the technical skills of the provider were the reason for their choice. Consistently, it was also found that for mild illnesses PFP were preferred. When a condition was considered serious then the main choice was a PNFP or a public facility.

Almost equal proportions of the clients of PFP, PNFP and public providers said that their choice was due to low costs of treatment. It seems then that in the areas surveyed cost was not a discriminating factor when choosing provider; public or private. Still, findings suggested that health care utilization had a social gradient as farmers sought care less often from private providers than traders who traditionally are better off than farmers. Also, less educated people tended to seek care less often than those with higher education. This is in line with a review of 48 sources of information on health care utilization in Uganda by Kiwanuka et al. in which it was found that socio-economic conditions were one of the determinants of health care utilization as the poor would seek care less often than the better off [15].

Previous studies from Sub-Saharan Africa show that proximity, skills of the provider, shorter waiting lines, longer opening hours, courtesy of the personnel, larger supply of health care personnel and pharmaceuticals and greater trust when seeking care for stigmatized diseases like tuberculosis, HIV/AIDS and other sexually transmitted diseases, are advantages of the private sector that patients appreciate [6,21-26]. For instance, a qualitative study from Kenya showed that self-treatment with drugs bought at shops or pharmacies are the main source of treatment of acute illness in both rural and urban areas, with the proportion of rural residents being slightly higher than urban. Private dispensaries were preferred over governmental because of lack of trust in governmental staff, poor interpersonal handling of patients at governmental facilities and a more speedy service at private health facilities [25]. A study from Uganda, comparing patients with sexually transmitted diseases in public and private health units, found that psychosocial factors such as attitudinal and normative beliefs influenced choice of provider more than socio-demographic factors [22].

This study documented that private providers form a large and versatile group of providers that offer services of shifting quality. They range from well organized institutions to one-person ventures where quality of care can be unsatisfactory. This may be particularly true for informal providers with no formal training. However, quality is also an issue among formal private providers even though they are required to have formal qualifications. Previous studies have demonstrated that the implementation of laws and regulations governing medical practice is poor in Uganda [27,28]. In a study from Uganda the authors conclude that in view of the large numbers of both formal and informal private practitioners, it is important to offer them monitoring and support to enhance quality of health care [29].

In this study, informal providers were shown to cover only a smaller fraction of the disease burden. While they outnumber all other types of providers combined, many of them practice part time and often without designated structures for their practice. A striking finding was that while traditional practitioners were the most numerous health providers in the studied communities, they are not the most popular choice for common illnesses. This could be a reflection of the limited range of services that they provide. However, it could also be an underestimation of actual use. People may not report their use of traditional practitioners as it may be seen as stigmatizing to use them due to various reasons, such as the popular belief that some traditional practitioners are associated with or possess witchcraft [24,30,31]. However, the same belief can also be a reason for not using those services other than when they are really called for. Other studies have shown that traditional practitioners see relatively few patients [32] and that they are primarily used for conditions such as mental problems or sexually transmitted diseases (Mudenda D, Lindfors A, Sundewall J, Wake W, Jonsson D, Forsberg BC. Clients of Traditional Health Practitioners in a Sub-saharan Setting - a Vulnerable Group with Complex Health Problems. Submitted.)

A large proportion of the interviewees resorted to self treatment when falling ill and a significant number of people did nothing at all about their illness. Together these two groups made up more than half of the responses to the illness. While partly due to mild illness, this could also reflect inadequate access to heath care. Even though a majority said that they self treated because they felt their problems were minor, a large proportion of them also said that the illness prevented them from attending work or school, indicating a more severe illness.

Self-medication may have negative health implications since many people use medicines that are available at home, maybe left over from earlier treatments. Studies have shown that a lack of knowledge of possible health risks and appropriate use of drugs exist among those who self-treat [33]. This highlights the potential dangers of self-medication such as over-dosage, inappropriate use of drugs and use of expired drugs. Further, incorrect treatment or no treatment when first falling ill might lead to longer disease episodes since time elapses before the patients get the right care [34]. It can also prolong the period during which an infectious disease can spread to others [35,36] and contribute to development of resistance to antibiotics in the community [37].

## Conclusions

This study has documented that private providers are multiple and reach a wide client base in rural Uganda. These providers offer an opportunity for improving access to care and equity [38]. However, private health care is versatile and the quality of private care, especially among informal providers, is many times unsatisfactory. Therefore, an appropriate policy and regulatory environment around private care providers must be established and guidelines need to be put in place to stimulate their appropriate training and monitoring. More knowledge on how the public and private sector can work together for improved health is needed in order to avail the opportunities, but also meet the challenges, that the private health care sector present.

## Competing interests

The authors declare that they have no competing interests.

## Authors' contributions

JKL was overall in charge of designing the study, the field work and the study report. He worked on the article through all its stages. SNG was the main field supervisor and contributed throughout to the study report and the article. AL drafted the first version of the article and contributed throughout on finalizing the article. SO was together with JKL in charge of the Uganda study. He worked on the study report and provided comments on the article throughout its course. VONN coordinated the study teams in the field, was one of the authors of the study report and provided inputs in drafting the article. BCF was the coordinator of the international Private Sector Programme in Health (PSP) of which this study was part. He was actively involved in setting up the programme, worked on tool development, organized the study and arranged for the study report to be converted into an article and worked on the article from its initiation to its completion.

## Pre-publication history

The pre-publication history for this paper can be accessed here:

http://www.biomedcentral.com/1472-698X/10/29/prepub

## References

[B1] De Costa A Diwan V Where is the public health sector? Public and private sector healthcare provision in Madhya Pradesh, India Health Policy 2007 84 2-3 269 76 10.1016/j.healthpol.2007.04.004 17540472

[B2] Levesque JF Haddad S Narayana D Fournier P Outpatient care utilization in urban Kerala, India Health Policy Plan 2006 21 4 289 301 10.1093/heapol/czl013 16790454

[B3] Prata N Montagu D Jefferys E Private sector, human resources and health franchising in Africa Bull World Health Organ 2005 83 4 274 9 15868018 PMC2626208

[B4] Ha NT Berman P Larsen U Household utilization and expenditure on private and public health services in Vietnam Health Policy Plan 2002 17 1 61 70 10.1093/heapol/17.1.61 11861587

[B5] Bustreo F Harding A Axelsson H Can developing countries achieve adequate improvements in child health outcomes without engaging the private sector? Bull World Health Organ 2003 81 12 886 95 14997241 PMC2572373

[B6] Osubor KM Fatusi AO Chiwuzie JC Maternal health-seeking behavior and associated factors in a rural Nigerian community Matern Child Health J 2006 10 2 159 69 10.1007/s10995-005-0037-z 16362233

[B7] Uplekar M Juvekar S Morankar S Rangan S Nunn P Tuberculosis patients and practitioners in private clinics in India Int J Tuberc Lung Dis 1998 2 4 324 9 9559404

[B8] Larson CP Saha UR Islam R Roy N Childhood diarrhoea management practices in Bangladesh: private sector dominance and continued inequities in care Int J Epidemiol 2006 35 6 1430 9 10.1093/ije/dyl167 16997849

[B9] Montagu D Franchising of health services in low-income countries Health Policy Plan 2002 17 2 121 30 10.1093/heapol/17.2.121 12000772

[B10] McPake B Hongoro C Contracting out of clinical services in Zimbabwe Soc Sci Med 1995 41 1 13 24 10.1016/0277-9536(94)00303-B 7667666

[B11] Gomez-Jauregui J The feasibility of government partnerships with NGOs in the reproductive health field in Mexico Reprod Health Matters 2004 12 24 42 55 10.1016/S0968-8080(04)24146-5 15626196

[B12] Loevinsohn B Harding A Buying results? Contracting for health service delivery in developing countries Lancet 2005 366 9486 676 81 10.1016/S0140-6736(05)67140-1 16112305

[B13] Birungi H Mugisha F Nsabagasani X Okuonzi S Jeppsson A The policy on public-private mix in the Ugandan health sector: catching up with reality Health Policy Plan 2001 16 Suppl 2 80 7 11772993 10.1093/heapol/16.suppl_2.80

[B14] Health Sector Support Programme II 2005 Kampala: Ministry of Health

[B15] Kiwanuka SN Ekirapa EK Peterson S Okui O Rahman MH Peters D Pariyo GW Access to and utilisation of health services for the poor in Uganda: a systematic review of available evidence Trans R Soc Trop Med Hyg 2008 102 11 1067 74 10.1016/j.trstmh.2008.04.023 18565559

[B16] McPake B Asiimwe D Mwesigye F Ofumbi M Ortenblad L Streefland P Turinde A Informal economic activities of public health workers in Uganda: implications for quality and accessibility of care Soc Sci Med 1999 49 7 849 65 10.1016/S0277-9536(99)00144-6 10468391

[B17] Okello DO Lubanga R Guwatudde D Sebina-Zziwa A The challenge to restoring basic health care in Uganda Soc Sci Med 1998 46 1 13 21 10.1016/S0277-9536(97)00130-5 9464664

[B18] Mbonye AK Hansen KS Wamono F Magnussen P Increasing access to prevention of mother-to-child transmission of HIV services through the private sector in Uganda Sex Transm Infect 2009 85 7 534 9 10.1136/sti.2009.037986 19703840

[B19] Mbonye AK Hansen KS Bygbjerg IC Magnussen P Intermittent preventive treatment of malaria in pregnancy: the incremental cost-effectiveness of a new delivery system in Uganda Trans R Soc Trop Med Hyg 2008 102 7 685 93 10.1016/j.trstmh.2008.04.016 18513767

[B20] Ensor T Cooper S Overcoming barriers to health service access: influencing the demand side Health Policy Plan 2004 19 2 69 79 10.1093/heapol/czh009 14982885

[B21] Brugha R Zwi A Improving the quality of private sector delivery of public health services: challenges and strategies Health Policy Plan 1998 13 2 107 20 10.1093/heapol/13.2.107 10180399

[B22] Nuwaha F Determinants of choosing public or private health care among patients with sexually transmitted infections in Uganda Sex Transm Dis 2006 33 7 422 7 10.1097/01.olq.0000204574.78135.9f 16531938

[B23] Msiska R Nangawe E Mulenga D Sichone M Kamanga J Kwapa P Understanding lay perspectives: care options for STD treatment in Lusaka, Zambia Health Policy Plan 1997 12 3 248 52 10.1093/heapol/12.3.248 10173406

[B24] Banda Y Chapman V Goldenberg RL Stringer JS Culhane JF Sinkala M Use of traditional medicine among pregnant women in Lusaka, Zambia Journal of alternative and complementary medicine 2007 13 1 123 7 10.1089/acm.2006.6225 17309386

[B25] Chuma J Gilson L Molyneux C Treatment-seeking behaviour, cost burdens and coping strategies among rural and urban households in Coastal Kenya: an equity analysis Trop Med Int Health 2007 12 5 673 86 10.1111/j.1365-3156.2007.01825.x 17445135

[B26] Ndulo J Faxelid E Tishelman C Krantz I "Shopping" for sexually transmitted disease treatment: focus group discussions among lay persons in rural and urban Zambia Sex Transm Dis 2000 27 9 496 503 10.1097/00007435-200010000-00002 11034523

[B27] Konde-Lule JK Okello DO Lubanga RN Arube-Wani J Legislatory framework for private medical practice in Uganda East Afr Med J 1998 75 9 544 8 10493059

[B28] Okello DO Konde-Lule JK Lubanga RN Arube-Wani J Lwanga J A review of regulatory framework for the emerging private health sector in Uganda J Clinical Epid 1998 51 suppl 1 S41 10.1016/S0895-4356(98)90134-2

[B29] Tawfik Y Nsungwa-Sabitii J Greer G Owor J Kesande R Prysor-Jones S Negotiating improved case management of childhood illness with formal and informal private practitioners in Uganda Trop Med Int Health 2006 11 6 967 73 10.1111/j.1365-3156.2006.01622.x 16772020

[B30] Kaboru BB Falkenberg T Ndubani P Hojer B Vongo R Brugha R Can biomedical and traditional health care providers work together? Zambian practitioners' experiences and attitudes towards collaboration in relation to STIs and HIV/AIDS care: a cross-sectional study Hum Resour Health 2006 4 16 10.1186/1478-4491-4-16 16846497 PMC1540435

[B31] Pilkington H Mayombo J Aubouy N Deloron P Malaria, from natural to supernatural: a qualitative study of mothers' reactions to fever (Dienga, Gabon) J Epidemiol Community Health 2004 58 10 826 30 10.1136/jech.2003.016089 15365107 PMC1763324

[B32] Filmer D Fever and its treatment among the more and less poor in sub-Saharan Africa Health Policy and Planning 2005 20 6 337 346 16155065 10.1093/heapol/czi043

[B33] Abdo-Rabbo A Household survey of treatment of malaria in Hajjah, Yemen East Mediterr Health J 2003 9 4 600 6 15748057

[B34] Adegboyega AA Onayade AA Salawu O Care-seeking behaviour of caregivers for common childhood illnesses in Lagos Island Local Government Area, Nigeria Niger J Med 2005 14 1 65 71 15832646 10.4314/njm.v14i1.37138

[B35] Needham DM Foster SD Tomlinson G Godfrey-Faussett P Socio-economic, gender and health services factors affecting diagnostic delay for tuberculosis patients in urban Zambia Trop Med Int Health 2001 6 4 256 9 10.1046/j.1365-3156.2001.00709.x 11348515

[B36] Sagbakken M Frich JC Bjune GA Perception and management of tuberculosis symptoms in Addis Ababa, Ethiopia Qual Health Res 2008 18 10 1356 66 10.1177/1049732308322596 18703818

[B37] Cars O Högberg LD Murray M Nordberg O Sivaraman S Lundborg CS So AD Tomson G Meeting the challenge of antibiotic resistance BMJ 2008 3371 337 10.1136/bmj.a1438 18801866

[B38] Patouillard E Goodman CA Hanson KG Mills AJ Can working with the private for-profit sector improve utilization of quality health services by the poor? A systematic review of the literature Int J Equity Health 2007 7;6 1 17 10.1186/1475-9276-6-17 PMC2186328 17988396

